# Monoclonal Antibodies in the Management of Inflammation in Wound Healing: An Updated Literature Review

**DOI:** 10.3390/jcm13144089

**Published:** 2024-07-12

**Authors:** Flavia Manzo Margiotta, Alessandra Michelucci, Cristian Fidanzi, Giammarco Granieri, Giorgia Salvia, Matteo Bevilacqua, Agata Janowska, Valentina Dini, Marco Romanelli

**Affiliations:** 1 Department of Dermatology, University of Pisa, 56126 Pisa, Italy; manzomargiottaflavia@gmail.com (F.M.M.); alessandra.michelucci@gmail.com (A.M.); giammarcogranieri@gmail.com (G.G.); giorgia.salvia2@gmail.com (G.S.); matteobevilacqua@ymail.com (M.B.); dottoressajanowska@gmail.com (A.J.); valentinadini74@gmail.com (V.D.); 2 Interdisciplinary Center of Health Science, Sant’Anna School of Advanced Studies of Pisa, 56127 Pisa, Italy; 3 Unit of Dermatology, Hospital of Carrara, 54033 Carrara, Italy; cri.fidanzi@outlook.it

**Keywords:** wound healing, monoclonal antibodies, chronic wounds, biologic therapy

## Abstract

Chronic wounds pose a significant clinical challenge due to their complex pathophysiology and the burden of long-term management. Monoclonal antibodies (mAbs) are emerging as a novel therapeutic option in managing difficult wounds, although comprehensive data on their use in wound care are lacking. This study aimed to explore existing scientific knowledge of mAbs in treating chronic wounds based on a rationale of direct inhibition of the main molecules involved in the underlying inflammatory pathophysiology. We performed a literature review excluding primary inflammatory conditions with potential ulcerative outcomes (e.g., hidradenitis suppurativa). mAbs were effective in treating wounds from 16 different etiologies. The most commonly treated conditions were pyoderma gangrenosum (treated with 12 different mAbs), lipoid necrobiosis, and cutaneous vasculitis (each treated with 3 different mAbs). Fourteen mAbs were analyzed in total. Rituximab was effective in 43.75% of cases (7/16 diseases), followed by tocilizumab (25%, 4/16 diseases), and both etanercept and adalimumab (18.75%, 3/16 conditions each). mAbs offer therapeutic potential for chronic wounds unresponsive to standard treatments. However, due to the complex molecular nature of wound healing, no single target molecule can be identified. Therefore, the use of mAbs should be considered as a translational approach for limited cases of multi-resistant conditions.

## 1. Introduction

The definition of a chronic wound applies to a break in continuity of the skin or a mucous membrane that exhibits difficulty to heal within an expected time frame. Although a unique time frame has not yet been defined, a chronic wound is described as a lesion that does not undergo spontaneous resolution within 12 weeks or does not show a tendency to heal within 3 months, despite proper wound management [1,2]. Chronic wounds have a great impact on a patient’s quality of life and represent a global socioeconomic issue, as it is estimated that about 1–2% of the population worldwide will develop chronic wounds during their life span [3,4]. Chronic wounds show a wide heterogeneity in terms of etiology and can be classified into typical and atypical ulcers [5]. The former type includes vascular ulcers (including those due to venous and/or arterial insufficiency), diabetic ulcers (neuropathic, arterial, or mixed), and pressure ulcers. Atypical ulcers include inflammatory ulcers, neoplastic ulcers, and those related to genetic predisposing factors, infections, radiation and medical exposure, and others [6,7]. Specifically, wounds developing as a consequence of immune system dysfunction include pyoderma gangrenosum (PG), vasculitis, and vasculopathies (such as cryoglobulinemia).

The physiological process of wound healing consists of a set of sequential and overlapping phases that begin with a hemostatic phase and subsequently proceed through inflammation, proliferation, and remodeling, where vascularization precedes the innervation process [8,9]. Chronic ulcers lose this linear organization, and different parts of the ulcer may be in different stages of healing, making it therefore inappropriate to use the same therapeutic approach for the entire ulcer. One of the major steps of healing is represented by wound inflammation, which manifests as a fine balance between defective and excessive inflammatory signals, where both result in delayed healing. In fact, if on the one hand inflammation represents a central component of healing, on the other hand, delayed wound closure is often caused by persistent inflammation that does not allow the wound to proceed into the proliferative phase [10]. Innate immunity represents the first-line, nonspecific defense against tissue damage, and its dysregulation is responsible for preventing tissue repair. Damaged keratinocytes are the first to respond to tissue damage, activating several pathways of inflammation involving the action of damage-associated molecular patterns (DAMPs) and pathogen-associated molecular patterns (PAMPs) [11]. Equally recognized is the role played by a subtype of myofibroblasts and fibroblasts, whose deregulation is also associated with impaired tissue healing [12]. Some of the main actors in wound healing are also represented by macrophages whose function is to phagocytose and eliminate necrotic tissue during wound remodeling [13]. Macrophages are classically divided into two phenotypes, with M1 producing pro-inflammatory cytokines such as tumor necrosis factor-alpha (TNF-α), interleukin-1 (IL-1), interleukin-6 (IL-6), interleukin-12 (IL-12), and interleukin-17 (IL-17) [14] and M2 playing an opposite anti-inflammatory role [15]. The regulation of macrophages’ polarization therefore represents a promising approach for the management of correct inflammatory balance in wound healing, and many strategies have been applied to restore the pool of M2 macrophages in chronic wounds [16]. Moreover, the persistence of neutrophils in the wound leads to the continuous degradation of collagen and delays the wound healing process [17]. It is also important to mention the emerging role of the adaptive immune response in the process of tissue healing. Indeed, γδ T cells are involved in the process of tissue repair. They have been located in the epidermis and dermis of excisional wounds in both mouse and human samples. In chronic wounds, they express IL-17, appear reduced in number, and demonstrate dysfunctional activity [18]. Furthermore, regulatory T (T-reg) cells and non-cytotoxic innate lymphoid cells (ILCs) play a key role in promoting tissue healing [19].

This complex molecular landscape of wound healing gives rise to clinical conditions that can often be resolved with a classic therapy based on advanced dressings. In particular, the standard therapy of chronic ulcers to date is based on the principles of Wound Bed Preparation (WBP) summarized with the acronym TIME [20]. Aside from local wound dressings, further treatments include ultra-specialized techniques such as the use of bioengineered tissue, photo biomodulation, and epidermal skin grafting [21,22,23]. The individualization of wound care is becoming increasingly feasible, in part due to the numerous applications of technology as a novel tool for the diagnosis, treatment, and prevention of chronic wounds [24,25,26,27]. Unfortunately, even with the optimization of basic wound care and appropriate management, certain chronic wounds persist and therefore need ulterior and more adequate clinical tools, which in some circumstances are not typical of the world of wound healing.

Monoclonal antibodies (mAbs) are immunoglobulins able to target a specific antigen epitope due to their variable domains on heavy and light chains. These domains are responsible for their high target specificity, and it is thanks to this specificity and low toxicity that these antibodies have rapidly gained a predominant role in the pharmaceutical industry, substantially displacing the use of small molecules [28]. In the dermatological field, the use of mAbs to target specific molecules involved in the inflammatory process is widely employed in different chronic inflammatory diseases such as psoriasis, atopic dermatitis, hidradenitis suppurativa, and PG [29,30,31,32,33]. Despite the widespread use of these molecules and the isolated yet distinct scientific evidence regarding their usefulness in the field of tissue repair, surprisingly, the current literature does not provide an overview on the current use of mAbs in wound healing. In fact, while there are many articles that report on the effectiveness of individual molecules, a paper that combines an overview of the main cytokines involved in the propagation of chronic wounds and the clinical results of mAbs that block these cytokines has yet to be published. This review focuses on the role of mAbs as an immunomodulatory therapy in chronic wounds recalcitrant to standard therapies. Each paragraph will illustrate the putative pathogenic role of single cytokines in chronic wounds, as well as the medical communities experience using those mAbs targeting the respective molecule. In particular, the role of inflammation in wound healing and its main protagonists will be discussed, examining how they are involved in the propagation of chronic wounds with a specific focus on those resulting from inflammatory dermatoses.

## 2. Materials and Methods

Our review was based on a search performed up to May 2024 on the PubMed, Google Scholar, Cochrane Skin, EBSCO, Embase, and MEDLINE databases. Research was conducted by using and matching the following terms: “wound healing”, “wounds”, “ulcers”, “biologics”, “pyoderma gangrenosum”, “adalimumab”, “infliximab”, “etanercept”, “brodalumab”, “ixekizumab”, “risanzikumab”, “guselkumab”, “secukinumab”, “tildrakizumab”, “ustekinumab”, “rituximab”, “anakinra”, “avacopan”, and “tocilizumab”. In the selected manuscripts, we included reviews, letters to the editor, real-life studies and case series, trials, and metanalyses. For each manuscript, we verified that the presented diseases were associated with actual ulcerative conditions. Manuscripts written in any language other than English were excluded from our analysis.

### 2.1. Eligibility Criteria and Study Selection

Inclusion criteria were based on (1) placebo- or active-comparator-controlled human studies; (2) trials evaluating the efficacy and safety of mAbs in wound healing; (3) case reports and case series on wounds treated with mAbs. Inflammatory conditions with marginal potential for ulcerative outcomes like hidradenitis suppurativa were excluded from the analysis since the reported efficacy of mAbs can be linked to the control of the pathogenetic immunological cascades of the disease even in the absence of ulcers.

### 2.2. Risk of Bias Selection

A revised Cochrane risk of bias tool for RCTs [34] was used by two investigators (F.M.M. and A.M.) to assess the quality of selected studies and to evaluate the overall risk of bias as low, high, or some concern. A third author (V.D.) was then consulted in the case of differing opinions.

## 3. TNF-α

TNF-α is a proinflammatory cytokine involved in the regulation of several cellular processes such as dermal fibroblast proliferation [35], the activation of endothelial cells [36], and the induction of keratinocyte adhesion [37]. The pathological role of TNF in wound healing derives from the finding that its levels are higher in the wound fluid of non-healing wounds compared to healing wounds [38]. Many studies focused on the relationship between TNF-α and venous leg ulcers, with a clear detection of TNF-α in intracapillary monocytes of venous ulcer biopsies [39] and increased levels of TNF-α on the margin of non-healing venous leg ulcers [40]. However, since there was no significant difference in the levels of bioactive TNF-α between the wound fluid of healing versus non-healing venous leg ulcers, it can be assumed that other inflammatory regulators are necessary to allow TNF-α to play its role as a mediator in wound healing [41]. Furthermore, a reduction in serum levels of TNF-α, parallel to the healing stage of the lesions, was observed in venous leg ulcers correctly treated with compression therapy [42]. In particular, other ulcerative conditions such as PG and Sweet’s syndrome show increased levels of TNF-α and its receptors in perilesional skin [43]. All this molecular evidence lays the foundation for the rationale of approaching the treatment of different ulcerative conditions using mAbs directed against TNF alpha, currently the most widely used mAb in the treatment of chronic wounds. Infliximab is an IgG mAb reported as a key therapeutic tool for the treatment of chronic wounds with different kinds of formulations. To date, the literature reports that subcutaneous (*sc*) injections have been used on complex idiopathic anal fistulas and lipoidica necrobiosis (LN); additionally, endovenous (*ev*) administrations (5 mg/kg) have been used for the treatment of refractory PG [44,45,46]. Several studies have reported the efficacy of the same *ev* dosage for the re-epithelization of ulcerated LN [47,48,49,50,51]. Topical applications of infliximab through a sterile hydroxyethyl cellulose gel have been used to treat refractory PG [52], while topical infliximab solutions (with subsequent application of an adhesive sheet) or in a gel formulation (under a hydrofiber dressing/an adhesive sheet) allowed a correct management of venous ulcers of the lower extremities [53]. Even adalimumab, a fully human recombinant IgG1 mAb against TNF-α, was used in the field of chronic wounds. In particular, the literature reports the clinical success of *sc* injections of adalimumab 40 mg (two vials at week (W) 0, one vial every 2 weeks) and elasto-compressive therapy, which demonstrated a reduction in the percentage of venous ulcers of the lower extremities [54]. Both weekly and bimonthly administration of adalimumab 40 mg reported good results in treating difficult cases of LN [55,56], even if Zhang et al. showed no improvement in an LN of the lower extremities and trunk treated with adalimumab, which then needed to be switched to etanercept [57]. Focusing on the treatment of PG, a recent review showed a greater efficacy of adalimumab compared to etanercept (75% vs. 61%) for the complete resolution of lesions [58], reconfirming the already established therapeutic role of adalimumab for the management of the disease [46,59,60]. Conversely, etanercept has been widely used for the management of NL [61,62,63,64], PG [65,66], and Behcet [67] lesions, mostly due to the demonstrated ability of Etanercept to decrease TNF-α activity in chronic wound fluid [68].

## 4. Interleukin-1 Inhibitors

IL-1 family members play a central role in the wound healing process. An alarmin function is displayed by both IL-1α and IL-1β, in which levels become higher immediately after a tissue injury and return to normal values at the end of the proliferation stage of wound healing [69]. Moreover, initial neutrophil recruitment to the site of injury, facilitated by IL-1 cytokines, contributes to the debridement process by inhibiting bacterial colonization [70]. As further confirmation of the role of IL-1 members in wound healing, it is known that both sporadic and syndromic PG cases present upregulated levels of IL-1α and -β [71,72]. A physiological modulator of IL cascades is represented by the protein IL-1 receptor antagonist (IL-1 Ra), which inhibits receptors of IL-1α and IL-1β and whose deficiency in animal models leads to delayed wound healing [73]. As further evidence, it is known that disruption of IL-1 signaling can improve the wound healing process by reducing scar formation [74,75]. For these reasons, IL-1Ra as a regulator of inflammation has been suggested as a target for the treatment of different kinds of refractory chronic wounds [76]. Anakinra is a recombinant human IL-1Ra that has been approved for the treatment of rheumatoid arthritis and neonatal-onset multisystem inflammatory disease. Low-dose anakinra in a gelatin–transglutaminase gel vehicle showed good results for the local treatment of diabetic wound healing [77], while different clinical case reports reported its efficacy in the treatment of PG with *sc* injections [58].

## 5. Interleukin-6 Inhibitors

IL-6 is a proinflammatory cytokine with an established role in wound healing as a chemoattractant for monocytes and neutrophils, thus directing the inflammatory phase of the wound process [78] (Komi 2020). The efficacy of IL-6 detection for the early diagnosis of wound infection was proposed, considering the ability of IL-6 to activate C-reactive protein (CRP), a well-known biomarker for evaluating infection status [79,80,81]. IL-6 is also able to stimulate re-epithelialization through the activation of STAT3-dependent pathways, which lead keratinocytes to respond to mitogenic factors that address migration [82,83]. Moreover, IL-6 acts on collagen production in dermal fibroblasts, so a putative role in the pathogenesis of autoimmune diseases such as systemic sclerosis has been suggested [84]. As a further confirmation, PG patients present high skin and serum levels of IL-6 and its receptor [72], with a significant reduction after correct treatment of the disease [85]. The use of tocilizumab, a humanized anti-human IL-6R mAb approved for rheumatoid arthritis, juvenile idiopathic arthritis, and Castleman disease, has been applied in the context of various autoimmune diseases presenting with skin ulcers. The current use of tocilizumab in wound healing is generally limited to the management of inflammatory conditions, including systemic sclerosis [86], Behcet’s syndrome [87], and systemic rheumatoid vasculitis [88]. Tocilizumab was used to treat mixed arteriovenous ulcers of the lower limbs with recurrent erysipelas in a patient affected by idiopathic multicentric Castleman disease (iMCD) [89]. Furthermore, two clinical cases also proved Tocilizumab’s efficacy in improving PG ulcers in patients affected by rheumatoid arthritis and Takayasu arteritis, respectively [90,91].

## 6. Interleukin-17 Inhibitors

IL-17 family members include six cytokines (IL-17A through IL17F) that are classically established as inflammatory actors of autoimmune diseases and whose role in wound healing is progressively being investigated. IL-17 is produced by dermal γδ T cells, which display a pro-reparative action in normally healing wounds, and on the contrary are found to be reduced and dysfunctional in chronic wounds [92]. This evidence would explain why mice deficient in IL-17 show delayed healing [93], but the role of IL-17 in the chronicization of wounds cannot only be linked to a possible reduction in cytokine levels. In fact, IL-17 is able to phosphorylate NF-κB and STAT3 pathways and subsequently promote the transcription of IL-1β in mouse keratinocytes, which is an established causative agent of impaired wound healing [94]. It is also known that the expression of IL-17 is significantly increased in keloid tissue, therefore linking it to the formation of exaggerated scar tissue and impaired wound healing [95]. For these reasons, an upregulation of IL-17 would be able to derail the physiological healing process; in fact, lesional biopsies of ulcerative conditions such as PG and Sweet’s syndrome show higher levels of IL-17 [43].

Even if laboratory evidence highlights a potential role of IL-17 in wound healing, few results are present in the literature on the clinical use of anti-IL-17 mAbs for the treatment of chronic wounds. The main data available are for the management of PG patients who were successfully treated with ixekizumab (anti-IL-17A/F), secukinumab (anti-IL-17A), and brodalumab (anti-IL-17 receptor) [96,97,98,99]. Local delivery of anti-IL-17 Ab for 3 consecutive days also showed accelerated physiological healing in a diabetic mouse model [100]; however, no data on human patients are yet available.

## 7. Interleukin-23 Inhibitors

IL-23 is a heterodimer constituted by a p19 subunit and a p40 subunit, which is shared with IL-12 [101]. IL-23 is a tight regulator of IL-17 expression in T cells and has been demonstrated to be a major determinant of macrophage polarization in skin wounds [102]. The blockade of IL-23p40 affects the modulation of wound healing by upregulating MMP-9, which is known to have a downstream role in angiogenesis [103]. In particular, IL-12/IL-23p40 knockout mice experienced accelerated oral mucosal wound healing thanks to an early inflammatory response and vascularization process [104]. Increased expression of IL-23 genes was observed in PG lesions [105]. This evidence may explain the numerous therapeutic successes of PG treatment with IL-23 inhibitors such as guselkumab (anti IL-23/p19), risankizumab (anti IL-23/p19), tildrakizumab (anti IL-23/p19), and ustekinumab (anti IL-12/23p40) [106,107,108]. Good efficacy of IL-23p19 mAbs was even shown through topical applications on full-thickness wounds of the dorsal surface of diabetic mice, leading to significantly improved wound re-epithelialization [102]. A recent paper published by our team also demonstrated the efficacy of risankizumab (*sc* injections of 150 mg at the start of treatment, at W4, and then every 10 weeks thereafter) in the management of multirefractory PG when provided with contemporary parallel optimal wound care management [109].

## 8. C5A Inhibitors

A classical role in inflammatory response is displayed by the complement system, with both C3 and C5 having a well-established role in the physiology of wound healing [110,111]. C3, produced by human keratinocytes [112], is cleaved into C5a, which is a strong chemotactic for monocytes and polymorphonuclear leukocytes, promoting neutrophil migration during acute inflammation through its receptors C5aR and C5L2/C5aR2 [113,114,115]. It has been shown that C5a receptor-deficient mice presented a more effective wound closure [116] and that bacterial defense, in the absence of C5a, would be more easily achieved through the formation of the membrane attack complex C5b-9 [117]. Chronic wounds such as those found in PG showed higher levels of C5aR1 and C5aR2 in lesional skin [115], with a persistent expression of the complement system and STAT4 even in diabetic non-healing wounds [118].

C5A receptor inhibitors have been used in some groups as a therapy for antibody-associated vasculitis (AAV) [119,120,121]. Three-hundred and thirty-one patients with ANCA-associated vasculitis were treated in a 1:1 ratio with avacopan, an orally administered anti-C5A agent, or with a tapering dose of oral prednisone. In addition, all patients were given cyclophosphamide or rituximab. Remission was calculated via the Birmingham Vasculitis Activity Score (BVAS), and it was observed in 72.3% of patients taking avacopan and in 70.1% of patients taking prednisone at W26. At week 52, sustained remission was reported in 65.7% of patients receiving the C5A inhibitor and 54.9% of patients receiving oral glucocorticoids. This study showed that compared to the steroid-based therapy, avacopan was noninferior at week 26 and superior in sustaining remission at W52 [120].

## 9. Conclusions and Future Perspectives

**Figure 1:**
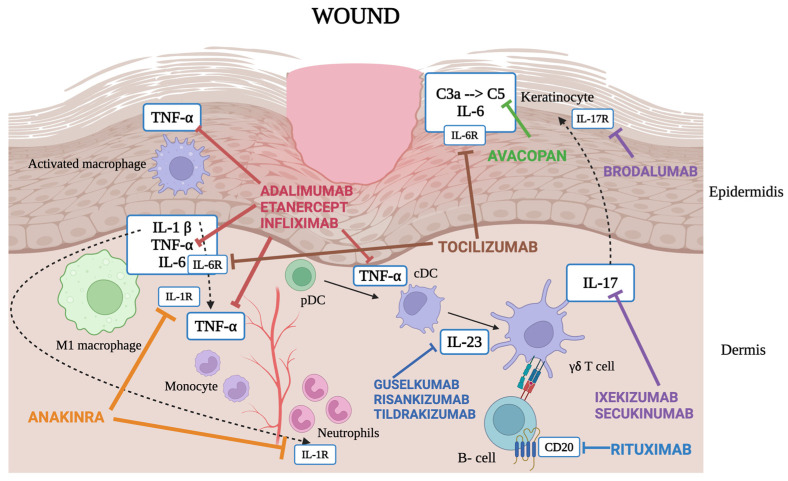
Comprehensive view of the main molecules involved in chronic wound pathogenesis and their targeted therapies. C: complement; CD20: cluster of differentiation 20; cDC: classical dendritic cell; IL: interleukin; IL-1R: interleukin 1 receptor; IL-6R: interleukin 6 receptor; pDC: plasmacytoid dendritic cell; TNF-α: tumor necrosis factor-alpha; γδ T, gamma delta T lymphocyte. Created with BioRender.com accessed on 24 June 2024.

Our work painted a complete overview of the successful use of various mAbs in the management of complex, recalcitrant chronic wounds, from expert teams all around the world. A portrait of the main molecules involved in the pathogenesis of chronic wounds and the therapeutic targets discussed in our paper is presented in Figure 1. The correct approach to chronic wounds that struggle to be managed with local medications still represents an open clinical challenge, and questions have been raised regarding the presence of an altered immunological background of ulcers recalcitrant to standard treatments. In particular, the necessity to deeper understand the pathophysiology arises from the need to optimize the type and number of therapies used for the management of ulcers, in consideration of the disproportionate costs that they generate in national healthcare systems. In particular, a 2017/2018 UK economic analysis revealed that the annual NHS cost of wound management was GBP 8.3 billion, of which GBP 2.7 billion and GBP 5.6 billion was linked to the management of healed and unhealed wounds, respectively [122]. Similarly, chronic wounds have been demonstrated to represent a significant economic burden on the healthcare system in Australia, with a total cost of chronic wounds estimated at AUD 3.5 billion annually [123]. Focusing on the total national health budget, Scandinavian countries reported that the costs of chronic wounds comprised 2–4% of the total healthcare expenditure [124]. From a general point of view, a treatment can be considered cost effective when it is economically advantageous in terms of both time and money. Understandably, rapid healing is a good strategy for containing the costs of treatment, and several studies reported that a higher cost per single medication may represent a smart solution if it leads to faster healing. In particular, Jemec et al. demonstrated that silver dressings resulted in more rapid chronic leg wound closure than wounds treated with non-silver dressings, therefore leading to a lower average total treatment cost per patient due to the shorter healing time despite the higher price per single medication [125]. The same results were achieved by Gilligan et al., who compared (1) becaplermin gel+ good wound care (GWC) versus (2) GWC alone in patients with diabetic foot ulcers, demonstrating that even if (1) was initially more expensive than (2), the former resulted in a more rapid wound healing and reduced the risk of amputation, thus gaining strength in overall long-term costs [126]. To date, in the field of wound healing, cost–benefit analysis studies of biological drugs have never been carried out, and there are still no comparative studies that quantify the reduction in the number of medications that are involved in the management of difficult patients. Our work represents the very first comprehensive view on the role of mAbs as a sensible therapeutic strategy in cases of refractory wounds. Further pharmacoeconomic studies are needed to verify the advantages that mAbs have considering the increased rapidity of wound closure and reduction in the number of advanced dressings needed. Similar studies conducted on rheumatoid arthritis showed that direct costs of biologic agents are significantly higher in comparison with traditional therapies, but if the analysis had included indirect costs (e.g., lower rate of hospital visits or orthopedic surgical admissions), biologics would have probably resulted in being less costly [127]. The authors also point out that a lower functional decline potentially associated with mAb therapy would lead to a reduced need for disability insurance, suggesting that health and disability insurances should be integrated in cost assessments [128]. The same reasoning can certainly be applied in the field of wound healing, where long-term complications represent a significant cost for national healthcare systems [129]. Moreover, results deriving from diseases that have historically benefited from biologics, such as IBD, showed that with a threshold of EUR 35,000/Quality-Adjusted Life Year, mAbs seem to be more cost effective than traditional therapies for the induction treatment of active IBD [130]. Our vision, motivated by these interesting results, leads us to encourage the field of wound healing to consider the advantages that mAbs can bring at a clinical and economic level in cases that are refractory to current standards of care. The key role of the various inflammatory cytokines and the interplay between epidermal and dermal cells is being systematically explored in the literature [131]. However, the inhibition of these cytokines does not always result in clinical resolution, perhaps due to the activation of a collateral pathway or the involvement of further pathogenic elements not yet explored. The biological drugs currently available on the market therefore represent an important therapeutic option in cases of recalcitrant ulcers, but we must consider that they were not originally designed solely for the field of wound healing. The main posologic options discussed in the previous paragraphs are reported in Table 1, while Table 2 summarizes the different wound etiologies and the various biologic treatments used for each of them.

It is crucial to emphasize that, if on the one hand molecular targets of biological therapy maintain an autonomous role in the pathophysiology of chronic wounds, on the other hand, they often represent a fundamental driver of many of the inflammatory dermatoses shown in Table 2. For this reason, the use of the proposed mAbs should be considered both in terms of the immunomodulatory action of biologics on the progression of wounds per se and the control of the pathogenetic immunological cascades of the underlying disease. The future goal therefore remains to further investigate the inflammatory cascades causing chronic ulcers, trying to direct the engineering of new molecules on the laboratory evidence that emerges. The creation of slow-release matrices or scaffolds of biological drugs also represents an interesting applicative perspective, for example those already in use for the release of PDGF-beta in mouse models of skin defects caused by diabetes [149]. The innovative use of scaffolds with anti-inflammatory properties has also been pursued by Qi et al., who designed a hybrid hydrogel with intrinsic immunomodulatory ability capable of increasing M1 to M2 transition and reducing multidrug-resistant Pseudomonas aeruginosa infection in diabetic foot ulcers [150]. The opportunity of including mAbs in wound dressings certainly represents an open challenge in the field of nanomaterials and biotechnologies, offering an interesting perspective for a wound healing target therapy. Our work has a few limitations. Despite having extensively elucidated the cytokines involved in wound healing and having provided current experiences on the use of specific mAbs, it is difficult to extract absolute considerations in terms of applicability and translate molecular evidence to the patient’s bedside. Firstly, the listed drugs must be used off label in most cases, and the bureaucratic procedures necessary to obtain approval often limit their use. Secondly, there are no studies to date that compare the effectiveness of various mAbs, so the number of experiences reported fundamentally depends on the marketing of the drug. It will be necessary to wait for future years to come to have numerically similar experiences and to be able to carry out an evaluation of the greater or lesser efficacy of mAbs in different etiologies. To conclude, even if the safety of mAbs has been widely explored in different fields [151], further studies on long-term efficacy and side effects are definitely needed to assess the validity of mAbs in chronic wound management, studies from which we believe will emerge evidence emphasizing the great fortune of being physicians today and having these therapeutic tools at our disposal.

## Figures and Tables

**Table 1:** Overview of the mechanisms of action, dosages, and formulations of the main drugs discussed in the review.

Drug	Posology	Target of Action
Adalimumab	80 mg *sc* W0, then 40 mg W1, then 40 mg every 1 or 2 weeks [54,55,56]	TNF-α
Anakinra	0.75 mg in a 3% gelatin–transglutaminase gel vehicle [77]	IL-1 receptor
	100 mg/d *sc* [58]	
Avacopan	30 mg *oral* twice daily [120]	C5A receptor
Brodalumab	210 mg *sc* weekly [99]	IL-17 Receptor
Etanercept	25 mg weekly *intralesional* [62] 25–50 mg *sc* twice weekly [61,65,66]	TNF-α
Guselkumab	100 mg *sc* monthly [106]	IL-23 (p19)
Infliximab	10 mg/mL (2 mL per lesion) *intralesional* on W0, 1, and 2 then 1-week treatment interruption; three treatment cycles [45] 100 mg in 5 mL saline, admixed to 15 g sterile hydroxyl ethyl cellulose gel [52] Solution (10 mg/mL) or gel formulation (0.45, 1, or 4.5 mg/g) subsequently covered with an adhesive sheet and a hydrofiber dressing for 24 h; application repeated after 3–4 weeks [53] Monthly *ev* 5 mg/kg [49,51] 5 mg/kg *ev* at W0, 2, 6, 12 [50], and 21 [47]	TNF-α
Ixekizumab	160 mg *sc* W0, followed by 80 mg every 2 weeks until W12, then 80 mg every 4 weeks [97]	IL-17A
Rituximab	1 gr *ev* W0, W2 [132] 375 mg/m^2^ *ev* once weekly [133]	CD20
Secukinumab	300 mg *sc* once a week for 4 weeks then 300 mg every 2 weeks until W32 [96]	IL-17A
Tildrakizumab	100 mg *sc* W0, W4, and then every 12 weeks [108]	IL-23 (p19)
Tocilizumab	8 mg/kg *ev* once a month for 6 months [86] 680 mg *ev* once a month [91] 162 mg *sc*, biweekly [90]	IL-6 receptor
Ustekinumab	90 mg *sc* W0, W4, then every 12 weeks [105]	IL-12/23 (p40)

*ev*: endovenous; IL: interleukin; TNF-α: tumor necrosis factor-alpha; *sc*: subcutaneous; W: week.

**Table 2:** List of monoclonal antibodies used for each disease.

Disease	Drug	Reference
Antibody-associated vasculitis	Avacopan	[119,120]
ANCA-associated vasculitis	Avacopan	[120,121]
Antiphospholipid syndrome	Rituximab	[134,135]
B-cell lymphoma	Rituximab	[133]
Behcet’s syndrome	Etanercept Tocilizumab	[67] [87]
Cryoglobulinemic vasculitis (HCV related)	Rituximab	[132,136,137]
Cryoglobulinemic vasculitis (non-HCV-related)	Rituximab	[137]
Complex idiopathic anal fistulas	Infliximab	[44]
Diabetic wounds	Anakinra	[77]
EGPA	Rituximab	[138]
GPA	Rituximab	[139,140,141,142,143,144,145]
LN	Adalimumab Etanercept Infliximab	[55,56,57] [57,61,62,63,64] [45,47,48,49,50,51]
PG	Adalimumab Anakinra Brodalumab Etanercept Guselkumab Infliximab Ixekizumab Secukinumab Rituximab Risankizumab Tildrakizumab Tocilizumab	[46,58,59,60] [58] [99] [58,65,66] [106] [46,52,146] [97] [98] [139,140,141,142] [107,109] [108] [90,91]
Rheumatoid vasculitis	Tocilizumab	[88]
Systemic sclerosis	Tocilizumab	[86]
Venous ulcers	Adalimumab Infliximab	[54] [53]
Vasculitis of the small vessels	Rituximab	[147,148]

ANCA: antineutrophil cytoplasmic antibodies; EGPA: eosinophilic granulomatosis with polyangiitis; GPA: granulomatosis with polyangiitis; LN: lipoid necrobiosis; PG: pyoderma gangrenosum.
